# A case of subclinical immune checkpoint inhibitor-associated myocarditis in non-small cell lung cancer

**DOI:** 10.1186/s12890-023-02417-4

**Published:** 2023-04-14

**Authors:** Yue Hu, Cuixia Liu, Shaojun Jin, Zihan Yi, Chao Wang, Xiaohong Pan, Huaqiong Huang

**Affiliations:** 1 Key Laboratory of Respiratory Disease of Zhejiang Province, Department of Respiratory and Critical Care Medicine, Second Affiliated Hospital of Zhejiang University School of Medicine, Hangzhou, 310009 Zhejiang China; 2 Cancer Center, Zhejiang University, Hangzhou, 310058 Zhejiang China; 3 Department of Respiratory and Critical Care Medicine, Songyang people’s Hospital of Zhejiang, Lishui, 323499 Zhejiang China; 4 Department of emergency, Zhuji people’s Hospital of Zhejiang, Zhuji, 311800 Zhejiang China; 5 Department of Radiology, Second Affiliated Hospital of Zhejiang University School of Medicine, Hangzhou, 310009 Zhejiang China; 6 Department of Cardiology, Second Affiliated Hospital of Zhejiang University School of Medicine, Hangzhou, 310009 Zhejiang China

**Keywords:** Immune checkpoint inhibitors (ICIs), Immune-related adverse events (irAEs), Myocarditis, Sintilimab, Lung cancer

## Abstract

**Background:**

Immune checkpoint inhibitors (ICIs) have been widely used in the treatment of cancer. Moreover, immune-related adverse events (irAEs) have become a new clinical challenge. ICI-associated myocarditis is a rare but fatal condition among diverse organ injuries, and early recognition and effective interventions are critical for patients.

**Case presentation:**

In this report, we present the case of a healthy 60-year-old male who was diagnosed with lung squamous cell carcinomas following chemotherapy and received ICIs. The patient presented with asymptomatic cardiac biomarker elevation followed by immune-related myocarditis. Fortunately, the patient achieved a good clinical result after receiving high-dose steroids. The treatment with ICIs was discontinued because of recurrent increases in troponin T.

**Conclusion:**

ICI-mediated associated myocarditis is an uncommon but potentially life-threatening adverse event. The current data suggest that clinicians need to be cautious about reinitiation in low-grade patients; however, further study of the diagnosis and treatment is necessary.

## Background

Immune checkpoint inhibitors (ICIs) are widely used in various cancers, including lung cancer and melanoma. Thus far, the blockade of cytotoxic T lymphocyte-associated protein 4 (CTLA-4), programmed cell death protein-1 (PD-1), or programmed death-ligand 1 (PD-L1) has been reported to enhance antitumor activity and improve survival in patients by inhibiting T-cell activation and function[1, 2]. Meanwhile, immune-related adverse events (irAEs) have become a new clinical challenge. While ICI-associated toxicities can affect a variety of organs involving the neurological, endocrine, pulmonary, gastrointestinal, cardiovascular, and renal systems, myocarditis is considered a rare but fatal complication [3, 4]. A multicentre study showed a 40% death rate in 131 patients suffering from ICI-related myocarditis [5]. Most reported patients with ICI-associated myocarditis appear to have severe disease but have been successfully treated with systemic corticosteroid therapy [6–11]. Here, we report the case of ‘subclinical’ ICI-associated myocarditis in a patient with lung cancer.

## Case presentation

**Fig. 1:**
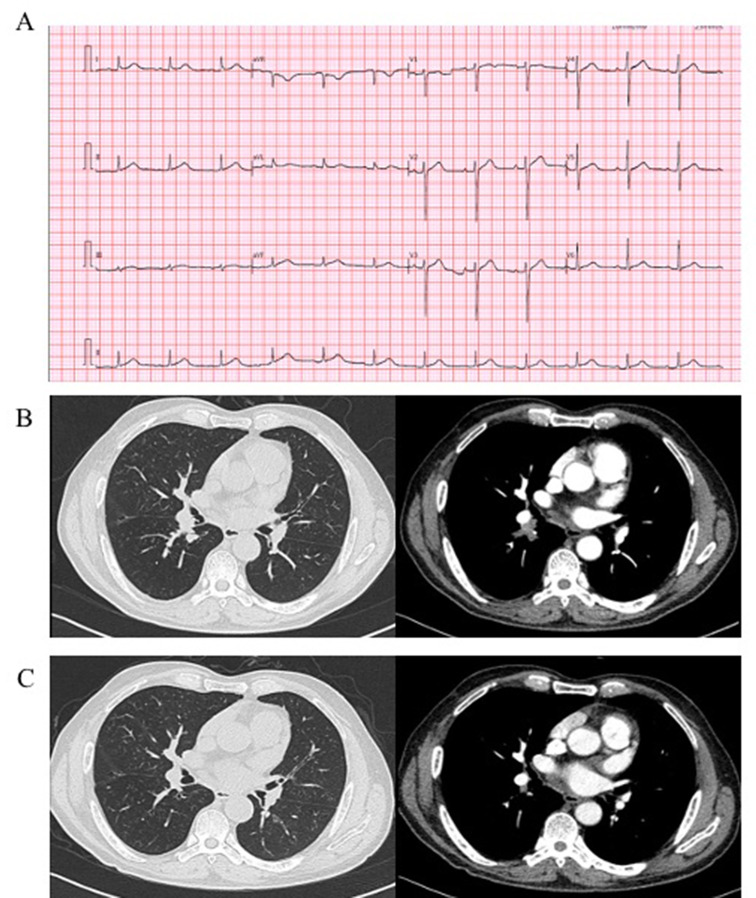
The examination of Electrocardiogram (ECG) and Chest CT in the patient with immune checkpoint inhibitor-associated myocarditis. (A) ECG showing normal sinus rhythm. (B) CT scan before the therapy. (C) CT scan after two cycle of chemotherapy with sintilimab

**Fig. 2:**
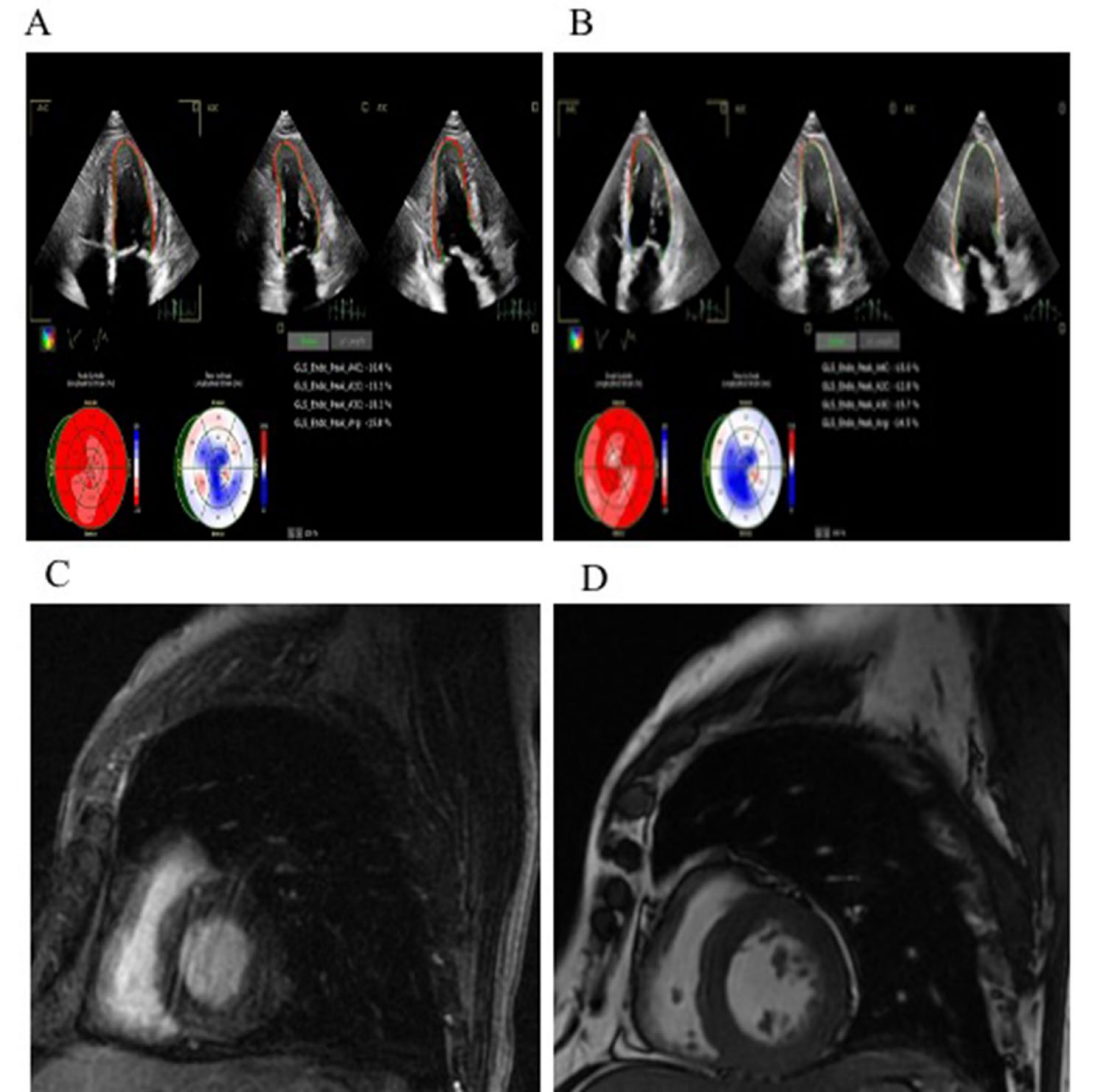
The cardiac ultrasound and cardiovascular magnetic resonance (CMR) in the patient with immune checkpoint inhibitor-associated myocarditis. (A) Cardiac ultrasound demonstrated the LV global longitudinal strain (GLS) was − 15.8%. (B) 20 days after treatment, the LV GLS improved to -14.5%. CMR imaging showed the patient without significantly late gadolinium enhancement (C) and T2 signal intensity (D)

A 60-year-old Chinese male with a history of diabetes, hypertension and coronary heart disease was diagnosed with lung squamous cell carcinomas T1cN0M0 Stage IA3. Consequently, sintilimab combined with carboplatin and albumin paclitaxel as neoadjuvant chemotherapy was given for two cycles from Sept 18th, 2021, to Oct 9th, 2021. After two cycles of the treatment, the patient presented with an increase in serum troponin T (TnT) of 0.303 ng/mL (Lab reference normal < 0.014 ng/mL) without obvious heart-related symptoms. The laboratory parameters are displayed in Table 1. Coronary artery computed tomography showed no evidence of acute coronary syndrome. Electrocardiogram (ECG) revealed a normal sinus rhythm without any ST-segment changes (Fig. 1A). Chest computed tomography showed that the lung lesions were significantly smaller than before, with no pulmonary congestion and no pleural effusion (Fig. 1B-C). Cardiac ultrasound showed a left ventricular (LV) ejection fraction (LVEF) of 67.6%, LV diastolic dysfunction (level II) and LV global longitudinal strain (GLS) of -15.8% (Fig. 2A). Cardiovascular magnetic resonance (CMR) imaging clearly showed neither late gadolinium enhancement (LGE) nor an elevated T2 signal intensity (Fig. 2C-D).

**Table 1:** The patient’s laboratory findings from starting point of immunotherapy (sintilimab) on hospital admission

Parameter	CK (U/L)	CK-MB (U/L)	TnT (ng/ml)	NT-proBNP (pg/ml)
Reference values	<164	<24	<0.014	<125
Baseline	61	7	0.014	217
Hospital admission	106	14	0.379	474
Day 1 after the steroids therapy	92	11	0.175	353
Day 2 after the steroids therapy	49	12	0.153	155
Day 11 after the steroids therapy	39	16	0.030	174
20 days after operation	107	5	0.188	2333

CK, creatine kinase; CK-MB, creatine kinase-myocardial band; TnT, troponin T; NT-proBNP, N-Terminal pro-brain natriuretic peptide

**Fig. 3:**
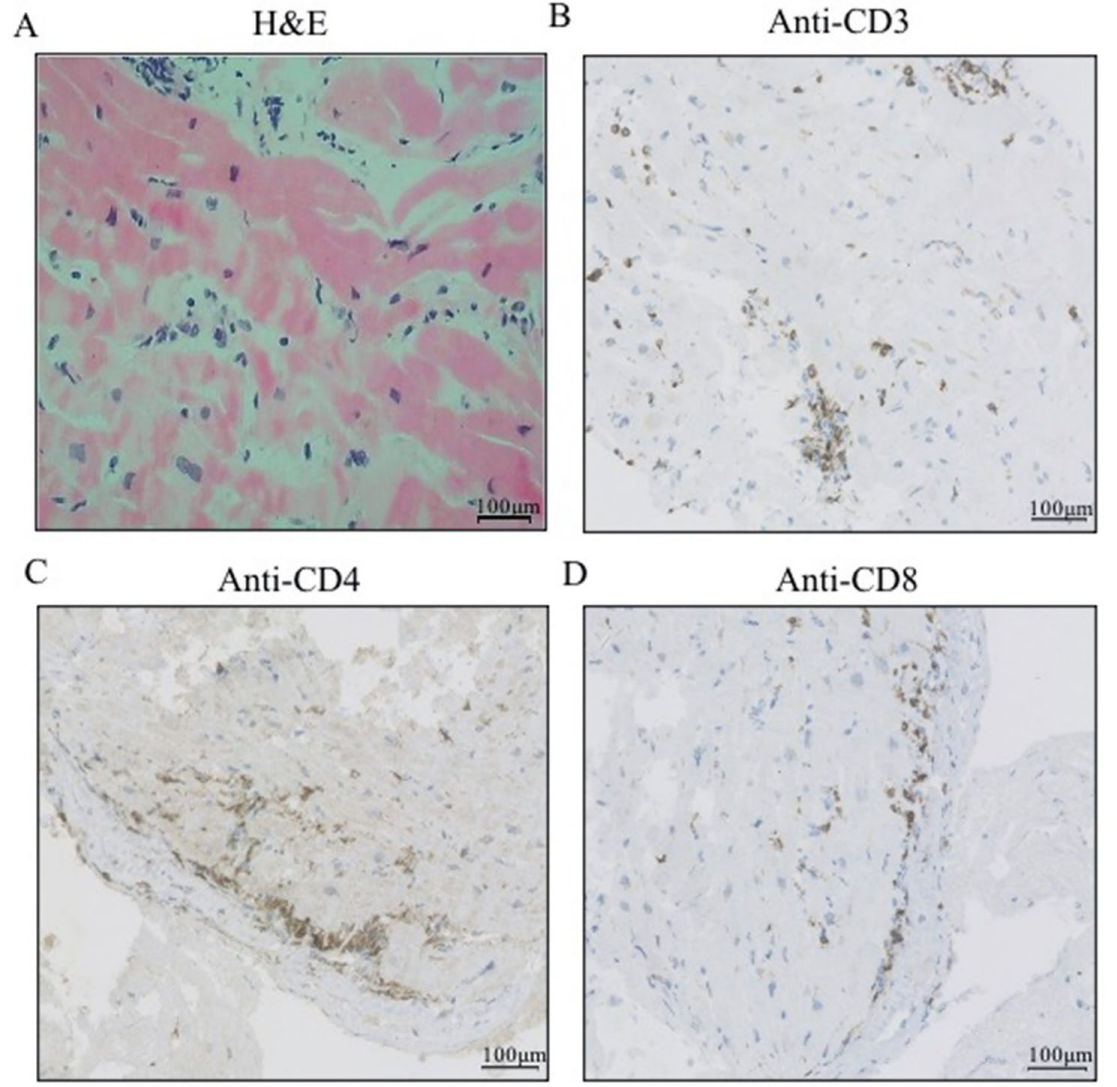
Histology (hematoxylin-eosin (H&E) and using anti-CD3, anti-CD4, anti-CD8) of myocardial biopsies with the indicated staining. (A) The myocardial biopsy stained with H&E revealed focal myocyte necrosis with multiple lymphocytic infiltrates. (B-D) Immunohistology detected numerous CD3^+^ T cells and proportions of CD4^+^ and CD8^+^ T cells. Scale bar: 100 μm

Based on the clinical presentation, laboratory results and medical history, we suspected immune checkpoint inhibitor (ICI)-associated myocarditis. The patient was given methylprednisolone (approximately 1 mg/kg/day) intravenously for 3 days, and then the dose was gradually decreased. The troponin T levels then decreased over the subsequent 2 weeks (Table 1). The patient received a right lower thoracoscopic pulmonary lobectomy on Nov 22nd, 2021. Twenty days later, the patient’s TnT was significantly elevated at 0.188 ng/mL, and his serum N-terminal pro-brain natriuretic peptide (NT-proBNP) was 2333 pg/ml (Lab reference normal < 125 pg/ml). Cardiac ultrasound showed a LVEF of 67.9%, LV diastolic dysfunction (level II) and a LV global longitudinal strain (GLS) of -14.5% (Fig. 2B). The myocardial biopsy showed degeneration of myocardial cells and an inflammatory infiltrate consisting of predominantly CD3^+^CD4^+^CD8^+^ cells in the myocardium (Fig. 3A-D).

## Discussion and conclusions

ICIs are being widely used in the treatment of lung cancer and can contribute to a significant improvement in cancer-free survival, and they are well known to induce a variety of irAEs affecting the pulmonary, gastrointestinal, renal, neurological and endocrine systems[12–17]. Although the incidence of ICI-associated myocarditis is 0.06-1.14% [18, 19], it has a high mortality rate of up to 46% [20].

Patients suffer from irAEs due to sintilimab therapy, which has proven to be effective in non-small cell lung cancer (NSCLC) [21, 22]. Sintilimab is a recombinant humanized monoclonal antibody that binds to programmed death-ligand 1 (PD-L1) and has a different binding site and potentially greater affinity against PD-1 [22, 23]. Myocarditis has not been reported as an irAE of sintilimab. This is similar to the patient in this case who was treated with sintilimab and who demonstrated characteristics of subclinical ICI-associated myocarditis after a diagnostic examination and therapeutic interventions.

The diagnosis of ICI-associated myocarditis is based on the drug history, clinical features, cardiac biomarkers, ECG, and imaging examinations [24]. The typical clinical presentations include chest pain, shortness of breath, dyspnea, etc. However, there may be only asymptomatic TnT elevation in the early stage of the disease, so early screening and close monitoring are required [24]. The cardiac biomarkers usually include CK, CK-MB, and troponin. Troponin can be of great value in the diagnosis of ICI-related myocarditis [25]. By endomyocardial biopsy on autopsy, patients who were treated with ICIs and diagnosed with myocarditis (associated with chemotherapy) had elevated levels of cTn (94%). In addition, 46% of these patients developed serious cardiac events, including complete heart block with haemodynamic instability, cardiac arrest, and cardiovascular death [26]. Mahmood et al. reported a 4-fold increased risk of myocarditis with a TnT of ≥ 1.5 ng/ml [27]. In fact, troponin elevation may indicate not only myocarditis but also myocardial injury from other causes, such as acute coronary syndrome (ACS), chronic kidney disease or pneumonia. The thorough clinical evaluation of a patient with suspected ICI-triggered myocarditis is important. Some patients have had increased troponin after ICI treatment, indicating some cardiac damage, but without obvious cardiovascular symptoms [28]. One single-central study found abnormal serum troponin I in 17.1% of patients without clinical implications [29]. Although elevated levels of cTn are not a specific indicator of cardiotoxicity induced by ICIs, they predict a poor prognosis and should be interpreted as an indication of adverse cardiac events [26]. Therefore, it is important to identify early and dynamically monitor the occurrence of myocarditis [30]. A baseline value of troponin may be helpful in cases of subsequent unclear symptoms or equivocal diagnostic examinations [31]. In addition, increased troponin (94%) and abnormal ECG (89%) can be found in these patients [32]. ECG showed atrial or ventricular arrhythmias, such as atrial premature contraction, ventricular premature contraction, atrioventricular block and ST-segment changes. Although a decrease in LV function is often found by TTE or CMR imaging, the LVEF is relatively preserved in the early stages of the disease. Currently, CMR imaging and histopathological findings are the gold standard for the diagnostic criteria [33, 34]. CMR has become the primary tool for noninvasive assessment of myocardial inflammation in patients with suspected myocarditis [35, 36]. The typical CMR findings in myocarditis include oedema and myocardial delayed enhancement sparing the subendocardial region in a nonischaemic distribution [37]. However, an absence of abnormal findings on CMR does not rule out myocarditis [38]. Some reports suggest CMR might be less sensitive to early stages of myocarditis, and fewer than 50% of patients had LGE, which was consistent with ICI-associated myocarditis, and 42% of patients had neither LGE nor an increased T2 intensity signal [39, 40].

Although myocardial biopsy is not considered a first-line diagnostic test because of its invasive nature, due to the risk of cardiac perforation, and the localized nature of the biopsy sample [6], it is still the gold standard for the diagnosis of ICI-associated myocarditis, with a pathological definition based on immune infiltration and the presence of myocyte death [41]. Myocardial biopsy should be considered in all patients suspected to have ICI-associated myocarditis because a timely and accurate diagnosis is of great importance to patients. Furthermore, cardiac histology can not only be diagnostic but may also influence prognosis [32]. If ICI-associated myocarditis is suspected, all patients should be further evaluated with additional diagnostic studies, which may include invasive testing, including myocardial biopsy or coronary angiography. Justification for myocardial biopsy is based on the impact on patients, especially given the high mortality rate and false-negative rates of CMR. Additionally, immunosuppressive therapy beyond glucocorticoids is needed for patients with ICI-related myocarditis. The diagnosis of myocarditis results in the discontinuation of ICIs, which may lead to cancer progression in these patients. Whether corticosteroid therapy affects sensitivity to myocardial biopsy is unknown. Therefore, biopsy should be performed as soon as possible, while the initiation of corticosteroid therapy should not be delayed. In our case, in which elevated troponin levels, a longitudinal LV strain and a history of immunotherapy suggested immune-associated myocarditis, biopsy should be considered to formally confirm the diagnosis. The primary cardiomyopathic effects of chemotherapy are caused by myocardial cell loss, necrosis, and apoptosis mediated by oxidative stress on the myocardium [42]. ICI-associated myocarditis (associated with chemotherapy) is caused by inflammatory cell infiltration in the myocardium [43–45]. In our case, inflammatory infiltration of predominantly CD3^+^CD4^+^CD8^+^ cells was observed in the myocardium.

Some clinical analyses demonstrated that myocarditis could present with asymptomatic cardiac biomarker elevation or pericardial diseases [17, 34]. Therefore, it is crucial for doctors to diagnose ICI-related cardiotoxicities early and discontinue ICIs immediately [33, 46]. Steroids and immunosuppressants are recommended in the treatment of ICI-associated myocarditis [47]. A previous study showed that lower doses of steroids were associated with higher residual troponin and major adverse cardiac event rates [27]. The patient in this case achieved a good clinical response after receiving high-dose steroids.

The treatment of patients with ICI-associated myocarditis has been largely based on expert consensus. It is not recommended to restart ICI therapy in patients with irAE-associated severe myocarditis (grade ≥ 3 toxicity) [48]. However, it is controversial whether ICI therapy should be restarted after the cardiotoxicity has resolved in patients with subclinical ICI-associated myocarditis. According to the American Society of Clinical Oncology guidelines, permanent cessation should occur in patients with grade 1 toxicity (abnormal cardiac biomarker testing including ECG) [49], while it has been proposed that some patients can have careful reinitiation of ICI therapy [50]. In our study, the treatment with sintilimab was discontinued, and the patient with subclinical myocarditis (grade 1 toxicity) was not rechallenged because of the elevated level of TnT. Several studies on ICI rechallenge have shown that it may have some clinical benefits for some patients. However, only a small number of patients who were treated with anti-PD-1 antibody were included in previous studies. Escudier et al. reported that ICIs were reintroduced in four patients without any recurrences [33]. Hasson *et al. successfully* demonstrated two patients diagnosed with low grade (I and II) who renewed therapy without recurrence and had improvement in their disease [50]. Another case series showed that two patients with subclinical myocarditis were reintroduced to immunotherapy following the normalization of cardiac parameters [47]. One of them discontinued ICI therapy because of troponin elevation [47]. The current studies have several limitations, including retrospective designs, small sample sizes, and selection bias. The reinitiation of ICI therapy in patients with subclinical irAE-associated myocarditis (grade 1–2) remains to be further investigated.

The patient was observed in the subclinical period when taking glucocorticoid treatment, so that the biomarkers of the heart were rapidly reduced. However, the patient had a slight elevation in troponin without symptoms or signs. Puzanov et al. and his colleagues recommend baseline (before ICI initiation) followed by weekly troponin monitoring. The significance of its association with myocarditis outcomes may be questioned in the absence of a randomized study [47]. Actually, major concerns exist about assessing troponin to detect asymptomatic myocarditis during irAE treatment [51]. The experience has come from only one case so far, and further studies about the precise diagnosis and treatment of subclinical ICI-associated myocarditis are necessary to guide clinical work.

We reported a case of asymptomatic myocarditis associated with the novel PD-1 inhibitor sintilimab. ICI-associated myocarditis is rare but fatal to patients, so it is essential to provide early preventive and therapeutic treatments. The irAE suggests that the baseline levels of cardiac parameters should be tested and closely monitored. In addition, the cautious reinitiation of ICI therapy in patients with subclinical irAE-associated myocarditis should be considered. Due to the rarity of ICI-associated myocarditis, more high-quality evidence is necessary to rely on in the future.

## Data Availability

The datasets used and analysed during the current study are available from the corresponding author on reasonable request.

## Abbreviations

ICIs: mmune checkpoint inhibitors
CTLA-4: T lymphocyte-associated protein 4
PD-1: programmed cell death protein-1
PD-L1: programmed death-ligand 1
irAEs: immune-related adverse events
TnT: serum troponin T
ECG: Electrocardiogram
LV: left ventricular
GLS: global longitudinal strain
CMR: Cardiovascular magnetic resonance
LGE: late gadolinium enhancement
NT-proBNP: N-terminal pro-brain natriuretic peptide
ACS: acute coronary syndrome
CK: creatine kinase
CK-MB: creatine kinase-myocardial band.

## References

[1] Wei SC, Duffy CR, Allison JP (2018). Fundamental mechanisms of Immune Checkpoint Blockade Therapy. Cancer Discov.

[2] Sharma P, Allison JP (2020). Dissecting the mechanisms of immune checkpoint therapy. Nat Rev Immunol.

[3] Hu JR, Florido R, Lipson EJ, Naidoo J, Ardehali R, Tocchetti CG (2019). Cardiovascular toxicities associated with immune checkpoint inhibitors. Cardiovasc Res.

[4] Johnson DB, Balko JM, Compton ML, Chalkias S, Gorham J, Xu Y (2016). Fulminant myocarditis with combination Immune Checkpoint Blockade. N Engl J Med.

[5] Wang DY, Salem JE, Cohen JV, Chandra S, Menzer C, Ye F (2018). Fatal toxic Effects Associated with Immune Checkpoint inhibitors: a systematic review and Meta-analysis. JAMA Oncol.

[6] Ganatra S, Neilan TG (2018). Immune Checkpoint Inhibitor-Associated Myocarditis. Oncologist.

[7] Inayat F, Masab M, Gupta S, Ullah W. New drugs and new toxicities: pembrolizumab-induced myocarditis. BMJ Case Rep. 2018, 2018. 10.1136/bcr-2017-223252 PMC5787012 29367376

[8] Laubli H, Balmelli C, Bossard M, Pfister O, Glatz K, Zippelius A (2015). Acute heart failure due to autoimmune myocarditis under pembrolizumab treatment for metastatic melanoma. J Immunother Cancer.

[9] Mahmood SS, Chen CL, Shapnik N, Krishnan U, Singh HS, Makker V (2018). Myocarditis with tremelimumab plus durvalumab combination therapy for endometrial cancer: a case report. Gynecol Oncol Rep.

[10] Matsuo K, Ishiguro T, Najama T, Shimizu Y, Kobayashi Y, Mutou M (2019). Nivolumab-induced myocarditis successfully treated with corticosteroid therapy: a Case Report and Review of the literature. Intern Med.

[11] Wang Q, Hu B (2019). Successful therapy for autoimmune myocarditis with pembrolizumab treatment for nasopharyngeal carcinoma. Ann Transl Med.

[12] Liu KL, Chen JS, Chen SC, Chu PH (2013). Cardiovascular toxicity of molecular targeted therapy in Cancer Patients: a double-edged Sword. Acta Cardiol Sin.

[13] Chen DY, Huang WK, Chien-Chia Wu V, Chang WC, Chen JS, Chuang CK (2020). Cardiovascular toxicity of immune checkpoint inhibitors in cancer patients: a review when cardiology meets immuno-oncology. J Formos Med Assoc.

[14] Duan L, Wang L, Wang H, Si X, Zhang L, Liu X (2020). Clinical diagnosis and treatment of immune checkpoint inhibitors-related endocrine dysfunction. Thorac Cancer.

[15] Wang H, Guo X, Zhou J, Li Y, Duan L, Si X (2020). Clinical diagnosis and treatment of immune checkpoint inhibitor-associated pneumonitis. Thorac Cancer.

[16] Zheng K, Qiu W, Wang H, Si X, Zhang X, Zhang L (2020). Clinical recommendations on diagnosis and treatment of immune checkpoint inhibitor-induced renal immune-related adverse events. Thorac Cancer.

[17] Lobenwein D, Kocher F, Dobner S, Gollmann-Tepekoylu C, Holfeld J (2021). Cardiotoxic mechanisms of cancer immunotherapy - A systematic review. Int J Cardiol.

[18] Mahmood SS, Fradley MG, Cohen JV, Nohria A, Reynolds KL, Heinzerling LM (2018). Myocarditis in patients treated with Immune Checkpoint inhibitors. J Am Coll Cardiol.

[19] Moslehi JJ, Johnson DB, Sosman JA (2017). Myocarditis with Immune Checkpoint Blockade. N Engl J Med.

[20] Salem JE, Manouchehri A, Moey M, Lebrun-Vignes B, Bastarache L, Pariente A (2018). Cardiovascular toxicities associated with immune checkpoint inhibitors: an observational, retrospective, pharmacovigilance study. Lancet Oncol.

[21] Liu X, Yi Y (2020). Recent updates on Sintilimab in solid tumor immunotherapy. Biomark Res.

[22] Hoy SM, Sintilimab (2019). First Global Approval Drugs.

[23] Wang J, Fei K, Jing H, Wu Z, Wu W, Zhou S (2019). Durable blockade of PD-1 signaling links preclinical efficacy of sintilimab to its clinical benefit. MAbs.

[24] Wang F, Sun X, Qin S, Hua H, Liu X, Yang L (2020). A retrospective study of immune checkpoint inhibitor-associated myocarditis in a single center in China. Chin Clin Oncol.

[25] Bonaca MP, Olenchock BA, Salem JE, Wiviott SD, Ederhy S, Cohen A (2019). Myocarditis in the setting of Cancer therapeutics: proposed case definitions for emerging clinical Syndromes in Cardio-Oncology. Circulation.

[26] Sorodoc V, Sirbu O, Lionte C, Haliga RE, Stoica A, Ceasovschih A et al. The Value of Troponin as a Biomarker of Chemotherapy-Induced Cardiotoxicity. Life (Basel). 2022, 12. 10.3390/life12081183 PMC9410123 36013362

[27] Sarocchi M, Grossi F, Arboscello E, Bellodi A, Genova C, Dal Bello MG (2018). Serial troponin for early detection of Nivolumab Cardiotoxicity in Advanced Non-Small Cell Lung Cancer Patients. Oncologist.

[28] Moslehi J, Lichtman AH, Sharpe AH, Galluzzi L, Kitsis RN. Immune checkpoint inhibitor-associated myocarditis: manifestations and mechanisms. J Clin Invest. 2021,131. 10.1172/JCI145186 PMC7919710 33645548

[29] Delombaerde D, Vervloet D, Franssen C, Croes L, Gremonprez F, Prenen H (2021). Clinical implications of isolated troponinemia following immune checkpoint inhibitor therapy. ESMO Open.

[30] Nakagomi Y, Tajiri K, Shimada S, Li S, Inoue K, Murakata Y (2022). Immune Checkpoint inhibitor-related myositis overlapping with myocarditis: an Institutional Case Series and a systematic review of literature. Front Pharmacol.

[31] Brahmer JRLC, Schneider BJ, Atkins MB, Brassil KJ, Caterino JM, Chau I (2018). Management of Immune-Related adverse events in patients treated with Immune checkpoint inhibitor therapy: American Society of Clinical Oncology Clinical Practice Guideline. J Clin Oncol.

[32] Lehmann LH, Cautela J, Palaskas N, Baik AH, Meijers WC, Allenbach Y et al. Clinical Strategy for the Diagnosis and Treatment of Immune Checkpoint Inhibitor–Associated Myocarditis. JAMA Cardiology. 2021,6. 10.1001/jamacardio.2021.2241 34232253

[33] Escudier M, Cautela J, Malissen N, Ancedy Y, Orabona M, Pinto J (2017). Clinical features, management, and outcomes of Immune Checkpoint inhibitor-related cardiotoxicity. Circulation.

[34] Palaskas N, Lopez-Mattei J, Durand JB, Iliescu C, Deswal A (2020). Immune checkpoint inhibitor myocarditis: pathophysiological characteristics, diagnosis, and treatment. J Am Heart Assoc.

[35] Grani C, Eichhorn C, Biere L, Murthy VL, Agarwal V, Kaneko K (2017). Prognostic value of Cardiac magnetic resonance tissue characterization in risk stratifying patients with suspected myocarditis. J Am Coll Cardiol.

[36] Aquaro GD, Perfetti M, Camastra G, Monti L, Dellegrottaglie S, Moro C (2017). Cardiac MR with Late Gadolinium Enhancement in Acute Myocarditis with preserved systolic function: ITAMY Study. J Am Coll Cardiol.

[37] Friedrich MG, Sechtem U, Schulz-Menger J, Holmvang G, Alakija P, Cooper LT (2009). Cardiovascular magnetic resonance in myocarditis: a JACC White Paper. J Am Coll Cardiol.

[38] Abdel-Aty H, Boye P, Zagrosek A, Wassmuth R, Kumar A, Messroghli D (2005). Diagnostic performance of cardiovascular magnetic resonance in patients with suspected acute myocarditis: comparison of different approaches. J Am Coll Cardiol.

[39] Friedrich MGSO, Schulz-Menger J, Marciniak H, Luft FC, Dietz R (1999). Contrast media–enhanced magnetic resonance imaging visualizes myocardial changes in the course of viral myocarditis. Circulation.

[40] Zhang L, Awadalla M, Mahmood SS, Nohria A, Hassan MZO, Thuny F (2020). Cardiovascular magnetic resonance in immune checkpoint inhibitor-associated myocarditis. Eur Heart J.

[41] Caforio AL, Pankuweit S, Arbustini E, Basso C, Gimeno-Blanes J, Felix SB (2013). Current state of knowledge on aetiology, diagnosis, management, and therapy of myocarditis: a position statement of the European Society of Cardiology Working Group on Myocardial and Pericardial Diseases. Eur Heart J.

[42] Bojan A, Torok-Vistai T, Parvu A. Assessment and Management of Cardiotoxicity in Hematologic Malignancies. Dis Markers. 2021, 2021:6616265. 10.1155/2021/6616265 PMC7875649 33613788

[43] Thibault C, Vano Y, Soulat G, Mirabel M (2018). Immune checkpoint inhibitors myocarditis: not all cases are clinically patent. Eur Heart J.

[44] Koelzer VH, Rothschild SI, Zihler D, Wicki A, Willi B, Willi N (2016). Systemic inflammation in a melanoma patient treated with immune checkpoint inhibitors-an autopsy study. J Immunother Cancer.

[45] Ananthan K, Lyon AR (2020). The role of biomarkers in Cardio-Oncology. J Cardiovasc Transl Res.

[46] Tschope C, Cooper LT, Torre-Amione G, Van Linthout S (2019). Management of myocarditis-related cardiomyopathy in adults. Circ Res.

[47] Puzanov I, Subramanian P, Yatsynovich YV, Jacobs DM, Chilbert MR, Sharma UC et al. Clinical characteristics, time course, treatment and outcomes of patients with immune checkpoint inhibitor-associated myocarditis. J Immunother Cancer. 2021,9. 10.1136/jitc-2021-002553 PMC8231054 34162715

[48] Neilan TG, Rothenberg ML, Amiri-Kordestani L, Sullivan RJ, Steingart RM, Gregory W (2018). Myocarditis Associated with Immune Checkpoint inhibitors: an Expert Consensus on Data gaps and a call to action. Oncologist.

[49] Brahmer JR, Lacchetti C, Thompson JA (2018). Management of Immune-Related adverse events in patients treated with Immune checkpoint inhibitor therapy: American Society of Clinical Oncology Clinical Practice Guideline Summary. J Oncol Pract.

[50] Peleg Hasson S, Salwen B, Sivan A, Shamai S, Geva R, Merimsky O (2021). Re-introducing immunotherapy in patients surviving immune checkpoint inhibitors-mediated myocarditis. Clin Res Cardiol.

[51] Spallarossa P, Tini G, Sarocchi M, Arboscello E, Grossi F, Queirolo P (2019). Identification and management of Immune Checkpoint inhibitor-related myocarditis: use troponin wisely. J Clin Oncol.

